# Family Member, Best Friend, Child or ‘Just’ a Pet, Owners’ Relationship Perceptions and Consequences for Their Cats

**DOI:** 10.3390/ijerph19010193

**Published:** 2021-12-24

**Authors:** Esther M. C. Bouma, Marsha L. Reijgwart, Arie Dijkstra

**Affiliations:** 1 Department of Social Psychology, University of Groningen, 9712 TS Groningen, The Netherlands; arie.dijkstra@rug.nl; 2 EthoPet, 6815 HG Arnhem, The Netherlands; marsha@purrdoctors.nl

**Keywords:** domestic cats, relationship, function, family, child, friend, cat welfare, human–animal bond

## Abstract

Describing the relationship with one’s cat in human terms might reflect an underlying anthropomorphic view of the relationship which might be associated with an owner’s behavior towards their cat and the cat’s living environment. Owners self-categorized the relationship with their cat as either a ‘member of the family’, ‘as a child’, ‘best friend’, or ‘a pet animal’. The extent to which owner- and cat-related factors influence these four relationship descriptions are examined in survey data of approximately 1800 cat owners. Differences in outdoor access, care during absence of the owner, and access to the bedroom are examined between the four relationship perceptions. The owner’s age and household composition, ideas about their cat’s equality, support, and dependency, and whether their cat is a pedigree were significantly associated with relationship description and explained 46% of the variance. Owners who perceive their cat as a child or best friend see their cat as loyal, empathetic, equal to family, and dependent on them for love and care. Their cats are less often left in the care of others, are allowed more often in the bedroom and have less often (unrestricted) outdoor access. Moreover, cats perceived as children are more likely to live in a multi-cat household. Our results provide insight in the factors that are related to different (anthropomorphic) perceptions of the human–cat relationship and how perceptions relate to the living environment of cats.

## 1. Introduction

Pets can fill a relational void in owners’ lives or be an addition to human social support networks [1,2,3,4,5,6]. Previous research shows that pet owners bond with pets as they would with family members [7,8,9] and that pets can occupy similar social niches as partners, family members, and children [10,11,12,13]. Relationships with cats are often strong and long-term [14,15]. Cats are appreciated for their adaptability to small residences, independence, and (presumed) ease of care [15]. Although most felid species are basically solitary, cats have been successfully domesticated as companion animals [16,17,18] and domestic cats are even capable of living with other cats under the condition that enough resources are available [19]. Cats can fulfil their owner’s need for support, company, love and care [3,20,21,22,23,24,25,26]. Moreover, many cat owners describe the relationship with their cat in human terminologies such as family, friend, or child [27,28,29] which highlights the ‘human’ roles cats can have in their owner’s life. The aim of the present study is to explore how an anthropomorphic perception of the human–cat relationship is related to the living environments of cats.

The human–animal bond is defined as ‘a mutually beneficial and dynamic relationship between people and other animals’ [30]. While many studies showed the beneficial effects of cats on their owners [31,32,33,34,35,36,37], the effect of owners on their cats has received less attention. If an owner’s anthropomorphic relationship perception is related to social expectations of the cat, this could influence the owner’s behavior towards the cat and its living environment. Anthropomorphism has been related to both negative [38,39,40] and positive [41,42,43,44] outcomes for animals. Anthropomorphism can influence feline welfare in a negative way when unrealistic expectations result in disappointment and misinterpretation of behavior results in punishment or inadequate care [45]. Anthropomorphic descriptions (such as family member, child, or friend) might be related to a higher tendency to interpret cat behavior from a ‘human’ point of reference, influenced by knowledge of human behavior and one’s own experiences [46,47] and may influence how owners handle their cats and arrange their living environment. 

We propose the idea that the perception of the relationship (e.g., expectations, satisfaction, attachment) with a cat is influenced by the (often unconsciously) assigned social function of the cat which is reflected in an anthropomorphic relationship description. Our theory encompasses two social functions a cat can provide for its owner: provision of companionship and social support and being an object to love, care, and nurture [24,47]. Even though social support experienced by animals does not encompass practical or financial support [48], the presence and/or specific behaviors of the animal can be perceived as supportive [49]. There is some evidence that people who feel closely connected to their pet have small social networks [50] and that lonely people are more likely to attribute socially supportive abilities such as ‘considerate’ and ‘sympathetic’ to animals [51]. Besides companionship and social support, cats provide their owners with a way to fulfil their natural and innate need to nurture and care. During our evolutionary past, human families lived in close social groups, comparable with other primates [52], and care to infants was, besides both parents, also provided by sisters, brothers, and grandparents [53,54]. As humans belong to the few mammals in which both sexes share the duties of parenting [55,56], caring behavior is expected by both men and women, although the tendency to care seems to be stronger in women [57,58]. Therefore, women may perceive their cat more strongly as a ‘care target’ compared with men, which may be revealed in a different perception of the relationship with their cat.

### 1.1. Characteristics of Owner and Cat

Previously, owners’ relationship satisfaction and level of attachment were found to be associated with an owner’s social (living) situation [25,59,60], gender [28,61,62], age [28,63], and educational level [28]. However, the social function of the cat, and hence the relationship perception of the owner, might not only be associated with characteristics of the owner but also by characteristics of the cat. The human–cat relationship can be directly influenced by the cat’s behavior. As mentioned by Ines et al. [64], social behaviors of the cat are of importance in both the development as well as the sustainment of the relationship. Several studies showed differences in owner-directed behaviors of cats depending on the owners’ age, gender, household composition [65,66,67], and breed of the cat [68]. Secondly, the human–cat relationship can indirectly be influenced by the cat’s appearance. Certain cat breeds (such as the Ragdoll, British Shorthair, and Persian) are often perceived as ‘cute’ due to their relatively small limbs and round faces. These ‘infant-like’ features can evoke innate feelings of protection, care, and affection [69,70,71]. Because of previous findings related to human–animal relationships and (possible higher need) for social support, companionship, care, and nurture, owners’ social living situation, gender, age, and educational level, as well as cat breed, are considered in this study.

### 1.2. Consequences for the Cat’s Living Environment

As mentioned, an owner’s influence on their cat’s living environment has not received as much attention as the other way around. Therefore, we examine three aspects of the cat’s living environment that are related to feline welfare and might differ depending on the owner’s relationship perception. We examine (a) outdoor access, (b) access to the owner’s bedroom and (c) care during the owner’s absence. First, access to the outdoors has both beneficial effects (e.g., less boredom and obesity) as well as risks (e.g., accidents, poisoning, infectious diseases (reviewed in Foreman-Worsley et al. [72]). In the Netherlands it is common for cats to have outdoor access (although often restricted in area or duration). The number of cats living in a household, the cat being a pedigree [72], and owner’s personality [73] are associated with allowing cats outside or not. Second, most cats thrive in a predictable, stable, and controllable environment [74,75]. Consistent access to the bedroom provides a cat with a continuously accessible place for a quiet nap or a safe hide out. A previous study in the Netherlands showed that two-thirds of pet cats are allowed in the bedroom [76]. Moreover, allowing a cat to sleep on one’s bed reflects a high level of intimacy. Previous research showed that cats are more often allowed to sleep with the owner when the owner is single or has no children [77] or when the owner is female [67,78]. Third, during a period of absence, owners need to make sure their cat is provided with water, food, and a clean litterbox. In the Netherlands, most owners leave their cats in the care of friends and neighbors. The quality of care might differ between neighbors, friends, and professional cat sitters. Some owners hire a professional cat sitter or bring their cat to a boarding facility. Although some cats do not seem to mind staying in an unfamiliar environment, most cats show less stress related behaviors in their own territory [79]. 

### 1.3. Aim of the Study, Theoretical Model, and Hypotheses

The aim of this exploratory study is two-fold: (1) to identify owner and cat-related factors that are associated with (anthropomorphic) relationship perceptions and (2) to explore if the living environment of cats differ according to relationship perception.

A large cross-sectional sample of Dutch cat owners was presented with four relationship descriptions: pet animal, family member, child, and best friend (of which the last three are considered anthropomorphic perceptions) and asked to choose which description fits best. We examined the influence of factors related to the owner (e.g., social living environment, age, gender, and education) and the cat (e.g., being a pedigree, being the owner’s first cat, and owner-directed behaviors) and owner’s perceptions of their cat (e.g., supportive, empathetic, loyal, dependent) on relationship description. Furthermore, we examined if the living environment of the cats (e.g., outdoor access and care during owner’s absence) differs between the four relationship descriptions. To guide our research, we constructed a theoretical model in which we hypothesize how each of the four perceptions is related to the social role of the cat and how this might influence the cat’s living environment. 

The perception of a cat as a ‘member of the family’ relates to a group with at least two members (the owner and cat), but more likely includes multiple members. The ‘ingroup’ shares the same environment and experiences, and the cat might be perceived as more or less equal to the other family members. We expect that these cats are less often limited in their whereabouts; both inside and outside the house. As other people are likely present, the cat does not function as the owner’s main source for company and social support. 

The perception of a cat as ‘a child’ implicates a non-equal relationship in which the cat is dependent on the owner for care and protection which might be observed in less (unrestricted) outdoor access. When absent, we expect that these owners bring their cat to a boarding facility or hire a professional cat sitter. This relationship perception is probably more prevalent among (single) female owners and owners of ‘cute’ pedigree cats. 

The perception of a cat as ‘best friend’ encompasses an equal and loyal relationship. As owner and cat are equal, we expect no restrictions inside or outside the house. We expect that this relationship type is related to a high level of (non-romantic) intimacy which is reflected in the fact that the cat often sleeps on the owner’s bed. We expect that this description will be more prevalent among owners who live alone and that the cat might function as an important source of support and companionship. 

Lastly, perception of a cat as ‘a pet animal’ excludes the cat from a ‘human’ social function. The cat is not equal, empathetic, or loyal similar to a human and neither is the cat seen as a provider of social support. The cat receives (necessary) care, either by the owner or neighbors but is less often taken to a boarding facility. We expect that these cats are able to freely roam outside but have no access to the bedroom. This relationship description is probably more often chosen by men than by women. The aforementioned hypotheses for each perception are summarized in Table S1 in the Supplementary Materials section. 

## 2. Materials and Methods

### 2.1. Recruitment and Procedure

Participants were asked to complete an online questionnaire created in Qualtrics. The link to the questionnaire was distributed through several Dutch cat-related online newsletters (‘De Carnivoor’ and Katten Kenniscentrum Nederland); a hard copy magazine (‘Oppas voor de Poes’); and via the social media accounts of the ‘Purrdoctors’ (Facebook and Twitter). The questionnaire was only available in Dutch and consisted of questions about the cat, the relationship with the cat, attribution of emotions to cats, recognition of emotions on photographs and questions about the owner. If participants owned more than one cat, they were asked to keep one specific cat in mind when answering the questions. The questionnaire was available between 19 November 2018 and 1 February 2019. The Institutional Review Board of the University of Groningen Faculty of Behavioral and Social Sciences reviewed and approved the research (PSY-1920-S-0164).

### 2.2. Relationship Categories

Participants were asked ’How would you describe the relationship with your cat? Please choose the option that fits best’. Participants could choose from one of the following five options: (1) ‘my cat is a pet animal’, (2) ‘my cat is part of my family’, (3) ‘my cat is like a child to me’, (4) ‘my cat is my best friend’, and (5) ‘it is difficult to describe the relationship with my cat’. The latter category was not included in the analyses.

### 2.3. Characteristics of the Owner

Owners’ demographic data (gender, age, educational level, and postal code) were collected (including the answer ‘prefer not to say’). Owners were asked if they work with cats professionally, and if so, in what manner (veterinarian, veterinary nurse, cat groomer, cat breeder, cat behavior consultant or other). The presence of other people and other cats (besides the target cat) in the household was also assessed.

### 2.4. Characteristics of the Cat and Living Environment

Participants were asked to indicate their cat’s gender, pedigree (yes/no), neuter status (yes/no), if the cat is their first cat (yes/no), if they got their cat as kitten (under 3 months of age, yes/no) and if the cat is allowed in their bedroom (yes/no). Owners who allowed their cat in their bedroom were asked if the cat often sleeps with them on the bed (yes/no). Outdoor access was first examined by yes or no. Owners who provide their cat with outdoor access were asked how the cat is able to go outside (‘when someone opens the door’, ‘through a cat flap that is closed sometimes’ or ‘through a cat flap that is always open’) and to what kind of area the cat has access (everywhere, balcony, fenced garden, or catio). Care during the owner’s absence was assessed by presenting the owner with the following options: paid cat sitter at home, boarding facility, friend/neighbor care at home, or not applicable, seldom leave the house. Owners could indicate multiple answers.

### 2.5. Social Behavior of the Cat towards the Owner

We asked owners if their cat sits on its own initiative on their lap (yes/no) and how their cat responds to petting and lifting: ‘Does your cat allow you to stroke her?’ and ‘Does your cat allow you to pick her up?’ Owners could choose from the following answering categories: ‘never’ (0), ‘sometimes’ (1), ’often’ (2), and ‘(almost) always’ (3). Because category ‘never’ was indicated by less than 5 people, we combined category 0 and 1, as well as answering categories 2 and 3, in two new categories ‘never/sometimes’ (0) and ‘often/(almost) always’ (1).

### 2.6. Indicators of the Cat-Owner Relationship

We wanted to examine how owners think about their cat in a relational context and used seven statements from the Owner–Pet Relationship Questionnaire [62]. We selected only those statements that were a reflection of the owner’s ideas about the cat and not the statements that are related to the owner (for example, we did not select the statement ‘I love my cat’ or ‘I do not like leaving my pet in someone else’s care if I go interstate or overseas’). Owners were asked to rate their agreement with seven statements: (1) ‘my cat knows when I’m upset and tries to comfort me’ (empathy), (2) ‘my cat relies on me for love and care’ (dependency), (3) ‘my cat has the same rights and privileges as other family members’ (equality), (4) ‘my cat is more loyal to me than the people in my life’ (loyalty), (5) ‘my cat helps me get through tough times’ (support), (6) ‘my cat enjoys my company’ (company), and (7) ‘my cat gives me a reason to get up in the morning’ (purpose). Statements were answered on a 5-point Likert answering scale (‘strongly disagree’ (1), ‘disagree’ (2), ‘neutral’ (3), ‘agree’ (4), and ‘strongly agree’ (5). If appropriate, the options ‘not applicable’ or ‘I do not know’ could be chosen but were not included in each indicator’s average score.

### 2.7. Statistical Analyses 

Pearson’s Chi-square analysis and analysis of variance (ANOVA) were used to examine differences between the four relationship perception categories. Spearman rank correlations were used to examine associations between the variables. Multinomial regression was used to examine which variables are associated with each relationship perception category. By means of Pearson’s Chi-square analysis, we examined if the cat’s environment differed according to the owner’s relationship perception category. In the case of significant group differences, contrast analyses were used to determine which categories significantly differed from one another. We used version 26 of the SPSS software package by IBM (Armonk, NY, USA) to analyze our data. We corrected for multiple testing by using the Benjamini–Hochberg procedure [80] and considered a *p*-value below 0.001 as statistically significant. 

## 3. Results

### 3.1. Response and Sample Description

In total, 2607 participants started the questionnaire, of which 1859 (71%) answered at least 85% of the questions. Since the participant characteristics were assessed at the end of the questionnaire, we could not examine if the 748 people who dropped out had specific characteristics regarding gender, age, and educational level, compared with the 1859 people who finished the questionnaire. The final sample consists of 1803 participants since the 56 (3%) participants who chose the relationship perception category ‘it is difficult to describe the relationship with my cat’ were excluded. Analyses of postal codes indicate that participants lived all over the Netherlands (see Figure S1 in the supplemental results section). Table 1 summarizes characteristics of the respondents and their cats. Table S2 in the supplemental section displays percentages, means, and standard deviations for all variables; for the total sample and separately for each of the four relationship categories (pet animal, family member, child, and best friend). Our sample is not entirely representative of the Dutch population of cat owners. Most cats in the Netherlands are owned by families with children [81] while 22 percent of our respondents live alone. Moreover, women and people with a high educational level are over-represented in our sample. 

### 3.2. Relationship Perception

**Figure 1:**
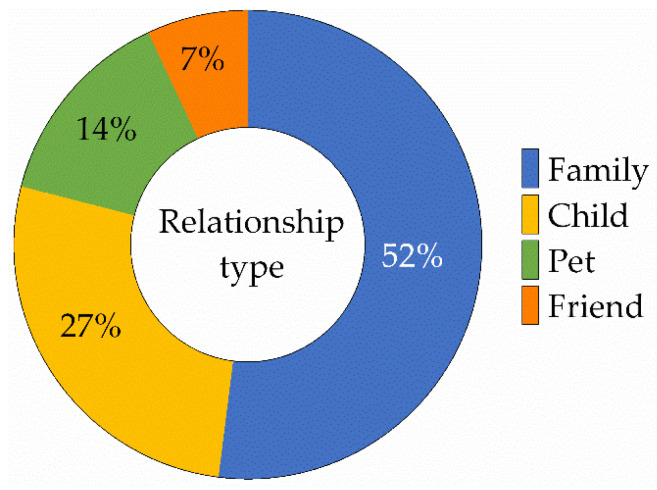
Percentage of participants for the different owner–cat relationship types (*n* = 1803).

The option ‘it is difficult to describe the relationship with my cat’ was not included in the analyses as it did not reflect a distinct relationship perception. Over half of the owners see their cat as part of their family (*n* = 943, 52%) and more than a quarter as their child (*n* = 493, 27%). Fewer owners see their cat as a pet animal (*n* = 258, 14%) and the lowest portion sees their cat as their best friend (*n* = 109, 7%). See Figure 1 for a graphical representation of these results. 

### 3.3. Determinants for Relationship Perception

The choice for relationship categories was associated with various variables as presented in Table 2. The multinomial regression analysis showed that the following variables were significantly associated with relationship description: owner’s age, presence of other adults in the household, the cat being a pedigree, and the relationship indicators equality, dependency, and support. The regression model was significant (Chi-square = 815,23, df = 138, *p* < 0.001), and together the significant factors explained 46% of the variance in relationship choice (Nagelkerke’s R^2^ = 0.463). Likelihood ratio test statistics are presented in Table 2. Neither gender, educational level, cat-related profession, company, purpose, nor being the owner’s first cat, owning the cat as kitten, or the cat’s gender was associated with the relationship perception category in this sample of cat owners.

#### Category Differences Regarding Significant Factors

To explore category differences between significant factors in more detail, pair-wise category comparisons were examined in post hoc analyses. Results of Chi-square and ANOVA tests are presented in Table S2. The most pronounced differences are present between owners who perceive their cat as a pet and owners who perceive their cat as child or friend. Owners who chose the relationship category ‘pet’ are older (more often above 55 years of age: 25% vs. 20% family, 14% child, 20% friend) and less often own a pedigree (17% vs. 31% family, 41% child, and 32% friend). Owners who chose either ‘friend’ or ‘child’ more often live alone (30% resp. 42% vs. 19% pet and 17% family). Additionally, owners who perceive their cat as a child are more often under the age of 35 years (44% vs. 19% pet, 27% family, and 30% friend) and more often own a pedigree cat (41% vs. 16% pet, 31% family, and 32% friend). 

Owner’s ideas about their cat’s dependency, equality, and support were significantly associated with relationship perception category choice. ANOVA analyses indicate that owners in the pet category agree the least with the statements, followed by owners in the family category, and owners in the friend and child category agree the most (Table S2). For all seven statements, differences between the pet category and the three other relationship perception categories are significant. Moreover, for all statements, except company, the level of agreement is significantly different between the family and child category. For none of the statements, significant differences were present between the child and friend category. 

### 3.4. Consequences for the Cat

Consequences for the cat’s living environment were examined with Pearson’s Chi-square analysis.

#### 3.4.1. Access to the Bedroom

**Figure 2:**
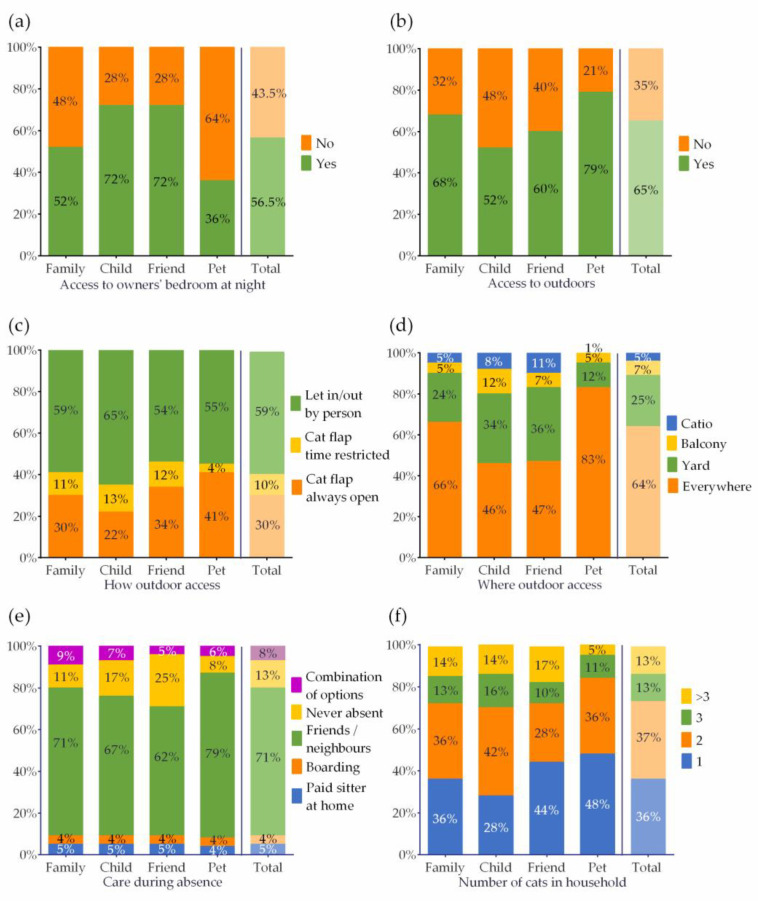
Aspects of the cat’s living environment. (**a**) access to owner’s bedroom at night, (**b**) outdoor access yes/no, (**c**) outdoor access how, (**d**) outdoor access where, (**e**) care during owner’s absence, and (**f**) multi-cat household, by relationship description.

Access to the owner’s bedroom (yes/no) differed significantly between the relationship categories (X^2^ (3, 1801) = 108.06, *p*-value < 0.001). Overall, 57% (*n* = 1017) of the cats in the sample are allowed in their owner’s bedroom. This percentage is higher for cats who live with owners who see them as child or friend (72%) and lower for cats who are perceived as family members (52%) or pet animals (36%) (see Figure 2a). Of the cats who are allowed in the bedroom, the majority (87%, 887 out 1017) sleep on the bed with the owner during (part of) the night. Although sleeping on the bed with the owner is slightly more prevalent in the child (91%) and friend (90%) group (vs. pet animal: 80% and family member: 87%) this difference was not significant after correction for multiple testing (X^2^ (3, 1010) = 8.81, *p*-value = 0.032).

#### 3.4.2. Outdoor Access

Outdoor access (yes/no) differed significantly between relationship perception categories (X^2^ (3, 1803) = 62.71, *p*-value < 0.001)). Outdoor access is most common in owners who see their cat as a pet (79%) and as a family member (68%) and less in owners who see their cat as a friend (60%) or a child (52%). See Figure 2b for a graphical representation of these results. Overall, two-thirds (65%) of the cats in this sample have access to the outdoors and for those cats (*n* = 1217), the how and where are examined. 

Overall, 30% of the cats that are allowed outside can go outdoors through a cat flap that is always open, 10% have a cat flap that is closed at certain times of the day, and 60% are only allowed outdoors when someone opens the door for them. Significant differences are present between the perception categories (X^2^ (6, 1170) = 24.87, *p*-value < 0.001). More cats that are seen as pets (41%) can go outdoors through a cat flap that is always open than cats that are seen as children (22%). See Figure 2c for a graphical representation of these results.

When we take a closer look at the outdoor area that the cats that are allowed outside can explore, we see that 64% of the cats can go wherever they want, while 25% are restricted to a fenced garden, 7% to a balcony, and 5% to a catio. Significant group differences are again present (X^2^ (9, 1120) = 79.00, *p*-value < 0.001). Cats that are seen as pet animals (83%) are most often allowed to roam freely, followed by cats that are seen as family (66%). Cats that are seen as friends or children least often are allowed to roam freely (47% resp. 46%). The latter (36% resp. 34%), on the other hand, are more often allowed outside in fenced gardens than cats that are seen as family (25%) and as pets (12%). The ratios for catio are the same as for fenced garden (friend 11%, child 8%, family 5%, pet 1%). Restriction of outdoor access to a balcony might be more a reflection of the living situation of the owner than the owner’s attitude towards their cat’s outdoor access. See Figure 2d for a graphical representation of these results.

#### 3.4.3. Care during Absence

Overall, 71% of the cat owners in our sample leave their cat in the care of friends or neighbors, 5% hire a sitter to take care of the cat at home, 4% bring the cat to a boarding facility, while 8% have no exclusive care protocol and indicate the use of combinations of the aforementioned options. Thirteen percent of the owners indicate that this question is not applicable as they do not leave the house for more than a couple of days. The division over these five categories differs significantly between the four perception groups (X^2^ (23, 1753) = 159.23, *p*-value < 0.001). Owners who see their cat as pets more often leave their cat in the care of friends and neighbors (79%) than the other three groups (family 71%; child 67%; and friend 62%). Owners who see their cat as friends (25%) or children (17%) more often indicated not to leave the house, compared with the owners who see their cat as a family member (11%) or pet (8%). The use of a boarding facility and cat sitter at home do not differ between the four perception groups. See Figure 2e for a graphical representation of these results.

#### 3.4.4. Other Cats in the Household

Our hypotheses did not encompass the presence of other cats in the household but as we found significant differences (X^2^ (9, 1799) = 45.52, *p*-value < 0.001) in the number of cats with regard to owner perception, the number of cats in the household is considered as a fourth aspect of the cat’s living environment. Owners in the child group have significantly more cats (Mean = 2.4, Sd = 1.7) than owners in the other three groups (pet: M = 1.9 (Sd = 1.7); family M = 2.3 (Sd = 1.8); and friend M = 2.3 (Sd = 1.9)). The proportion of only cats is highest in the pet group (48%, vs. family 36%; child 28%; and friend 44%). See Figure 2f for a graphical representation of the number of cats as indicated by four categories (one cat, two cats, three cats, and more than three cats) for each perception group.

## 4. Discussion

The aim of this exploratory study was to identify some of the determinants that influence (anthropomorphic) perceptions of relationships with cats and explore if living environments of cats differ accordingly. We showed in a large sample of Dutch cat owners that the majority of owners described the relationship with their cat in human terminologies, which suggests that owners see their cat as substitutes or additions to relationships with people. Moreover, we showed that the owner’s age, number of adults in the household, the cat being a pedigree, and the owner’s ideas about their cat’s equality, support, and dependency were significantly associated with relationship description and that these factors explained 46% of the variance. Lastly, we showed that the living environments of cats differ according to perceptions of their owner. 

We asked the cat owners to choose from four predefined perception categories that were based on the literature: pet, family member, child, and best friend. Only 3% of the cat owners did not recognize one of these categories as applicable. More than half of the owners in our sample perceive their cat as a member of the family, which is consistent with findings by others [27,42,82]. About a third describe the relationship with their cat in even more intimate terms, i.e., best friend or child. 

### 4.1. Determinants for Relationship Perception

The multivariate analyses showed several significant associations between the factors under study and relationship category. Consistent with other studies, we showed that characteristics of the owner have stronger associations with the perception of the human–cat relationship than characteristics of the cat [67,68,83,84]. Nevertheless, one cat characteristic, namely, being a pedigree, was related to owner’s ideas about their cat. Regarding the owner, only age and social living situation were significantly associated with relationship perception, which is comparable with a recent study by Ines et al. [64] that showed that gender was not related to relationship perception; however, age and household composition were. Other studies showed a positive correlation between relationship duration and attachment strength [6,85], but having the cat since kittenhood (as a proxy of relationship length) did not determine relationship perception in our study. 

As hypothesized, people who live alone more often see their cat as child or best friend, while people who are part of a couple or a family with children more often see their cat as family member or pet. Moreover, our data show a tendency for a gender-by-social environment effect, as single housed women more often report to see their cat as a child while single housed men more often report to see them as best friend. This is consistent with gender roles and stereotypes of women being more ‘caring’ than men. 

The multivariate analysis showed that a substantial proportion of the variance in relationship category choice was explained by a limited number of core factors. However, because several of the independent variables are related to each other, these omnibus analyses may obscure interesting and relevant associations of several variables with the relationship categories. Therefore, we will discuss the univariate findings in the light of our hypothesized associations that we formulated for each of the four relationship perceptions in our theoretical model.

### 4.2. Cats as Family Members

Overall, owners who perceive their cat as a family member were more often women than men, more often people under the age of 35 years, less often living alone, and more often owners of their (first) (pedigree) cat. Our finding that a higher proportion of women than men chose family member or child, might be explained by the fact that women have a stronger tendency to care for infants and other family members [58,59]. As expected, owners that see their cat as a family member agreed largely that the cat is equal to other family members, but not as much as owners who perceive their cats as best friend or children. The social role of the cats in the family group is limited, indicated by medium scores for loyalty, company, empathy, and social support. As hypothesized, cats of owners who see them as family have more outdoor access as two-thirds are free to roam outside and only a quarter are restricted to a fenced garden. We expected that these cats would also be allowed access to the bedroom, but they were overall less likely to be allowed in the bedroom than cats that are perceived as a friend or child. This might be explained by the observation that owners that see their cat as a family member rely less on their cat for company and support than owners that see their cat as a child or best friend. Another explanation might be that families (with children) are more concerned about bedroom hygiene, especially when their cat has access to the outdoors. 

### 4.3. Cats as Children

As hypothesized, owners who perceive their cats as their children are often young women under the age of 35, living alone, and are often owners of multiple (pedigree) cats. Young, single, and childless women might project their need to care on their cat(s). Supporting this hypothesis, identification as ‘a cat mom’ was found to be associated with the absence of human children in a small sample of women of reproductive age [29]. It has also been shown that pets can serve as an outlet for nurturing, especially for people without children [26]. However, as we have no information on the presence of children in the household, we can only speculate whether a cat that is perceived as ‘a child’ is actually being a substitute for a human child. 

Our findings can be (partly) explained by characteristics of the cat. Especially younger owners (who were more likely to see their cat as a child) indicated to own their cat(s) since kittenhood. The cuteness of the kitten(s) might have evoked feelings of protection in these young owners [69,70,71]. This effect might be enhanced by the fact that pedigree cats were over-represented in the ‘child’ group. Pedigree cats are often perceived as ‘cuter’ and ‘precious’ due to their ‘baby-like’ features and (often) high monetary value’.

We expected that owners in the child group would show a high tendency to care for and protect their cats, which was indeed reflected in owners’ agreement with statements about their cat being dependent on them for love and care, the fact that their cat is rarely left alone, less likely to be allowed outdoors, and when allowed outside often is restricted to a fenced garden or catio. These findings can partly be explained by the fact that pedigree cats are over-represented in the child group, as pedigree cats are less often allowed to roam freely due to their high monetary value [72]. However, restricting the cat’s whereabouts might also be a reflection of the social importance of the cat for its owner, as others found an association between strong attachment and less outdoor access [8]. Personality may also play a role, as neuroticism in cat owners is related to less outdoor access for their cat(s) [73]. In the human parent–child relationship, neuroticism is associated with a more authoritarian, overprotective caretaking style, and related to increased obesity and behavior problems in children [86]. High levels of neuroticism are also related to high levels of affection and anxious attachment of the owner towards the pet [87]. As neuroticism seems to be more common in women than in men [88] and women are over-represented in the child group, these findings might (partly) explain our results. 

The hypothesis that owners in the child group would less often leave their cat in the care of neighbors and friends, but instead would hire a professional cat sitter or bring their cat to a boarding facility was not supported by our data. Interestingly, just as in the friend group, a relative high proportion of owners in the child group indicated to never leave the house for multiple days (and therefore do not need someone to care for their cat). This could reflect an anxious or neurotic personality and/or a small social network. Alternatively, this group of owners might enjoy the company of their cat(s) so much that their need to go out might be lower compared with that of other cat owners. However, as we did not assess these possible explanations, we cannot formulate conclusions about this. 

People in the child group owned, on average, more cats than people in the other groups, which might be explained by a higher proportion of women in the child group, as women tend to own more cats than men [89,90]. Although domesticated cats are capable of living in close proximity to each other [19,91], the presence of other cats in their living environment can be pleasant or stressful, depending on the number of cats, the size of the area, access to food and other resources (litter boxes, water bowls, scratching posts, places to sleep/hide out), and the quality of the relationships between the cats [92,93]. As owners that see their cats as children less often provide outdoor access, the welfare of these cats might be compromised when the indoor environment is suboptimal (small, limited resources and absence of environmental enrichment such as food puzzles or (scented) toys) [94,95]. Although the categorization of relationship perceptions is not one-on-one comparable between studies, our results are concordant with Ines et al. [64] who showed that the number of owned cats and outdoor access are associated with relationship perception.

### 4.4. Cats as Best Friends

Owners who perceive their cat as their best friend are (as hypothesized) slightly more often male, often live alone, and often own only one (non-pedigree) cat. For this group of owners, we also expected high levels of equality, empathy, and loyalty and the cat being an important source of company and social support. Our results support the hypotheses on equality and support, but not for empathy and loyalty (the latter two did not determine relationship perception). We hypothesized that cats that are perceived as a best friend would more often be allowed outdoor access than the cats that are seen as a child. Contrary to this expectation, only 59% of these cats were allowed outdoor access, often in a fenced garden or catio. 

We also hypothesized that the perception of a cat as best friend would be reflected in allowing the cat access to the bedroom and to sleep on the bed. The ‘best friend’ cats were indeed more likely to be allowed in the bedroom, but we did not find differences between the relationship categories regarding sleeping on the bed. As owners that see their cat as a best friend seem highly attached to their cats, indicated by the highest levels of agreements on statements about their cat, our results are in concordance with Martens et al. [28] who showed a positive association between high levels of attachment and access to the owner’s bedroom at night. Being allowed in the bedroom might also be a consequence of the owner living alone, as found previously by Albert and Bulcroft [60]. In our sample we also found that, regardless of relationship perception, living alone was associated with access to the bedroom. This might be due to the absence of a partner (protesting against the presence of the cat) and/or a higher need for company. The number of cats in our study that are allowed in the bedroom is comparable with Overgaauw [76] who found that around two-thirds of Dutch cats are allowed in the bedroom. 

With regard to care during absence, the likelihood that the owner does not leave the house for several days is highest in this group of owners. Possibly, this group of owners has a smaller social network than owners in the other groups and therefore has less incentive to go away or does not know who to ask to care for their cat(s). As mentioned, when discussing our results in the child group, an alternative explanation might also be that owners in the friend group enjoy the company of their cat(s) so much that the need to go out is lower compared with other cat owners. However, we did not ask follow-up questions regarding why a respondent chose a specific option and therefore can only speculate.

### 4.5. Cats as ‘Just Pet Animals’

Owners that did not choose an anthropomorphic description of the relationship with their cat, but indicated that they see their cat as ‘just a pet’ are slightly more often male, do not live alone, are older than 35 years, and often own only one (non-pedigree) cat. As hypothesized, owners who perceive their cats as a pet animal (in Dutch ‘huisdier’ (‘house animal’)) do not see them as similar to humans as they agreed the least with statements about their cat’s equality. The low agreement scores for support, companionship, and purpose confirmed the limited social function of these cats. As hypothesized, cats that are seen as ‘just pets’ are least restricted in their outside access and whereabouts; they are often cared for by friends or neighbors when the owner is away and are less often allowed in the owner’s bedroom. It is important to note that this does not necessarily mean that the cat receives suboptimal care. When food and water are provided, the litterbox is scooped daily, and (for the cats who appreciate this) playtime and attention are given during absence of the owner, cats might be best off in their own familiar environment. Although these cats might not be allowed in their owner’s bedroom, they (often) have the possibility to go outside to escape something scary or stressful (such as a vacuum cleaner or visitors) that is present indoors.

### 4.6. Living Alone, Loneliness, and the Cats’ Sociocognitive Abilities 

Owners in the child and best friend group had a higher likelihood of living alone. It is tempting to say that cats of these owners function as additions or substitutes for human relationships, but as we did not did not assess the level of social support provided by people and feelings of loneliness, we cannot make firm statements about this. Interestingly, scores for the cat’s empathy were not significantly different between owners who do and do not live alone, which contradicts previous findings [37,96]. This could be due to the formulation of the ‘empathy’ statement in our study as ‘my cat knows when I am upset and tries to comfort me’, which actually includes two statements (‘knows when upset’ and ‘trying to comfort’). This probably confused the respondents, reflected by a relatively high percentage of respondents (4%) that selected the option ‘I do not know’ (compared with 1–2% for the other statements). 

Attribution of sociocognitive qualities such as ‘perceptive’, ‘empathetic’, and ‘considerate’ to animals makes it possible for them to be a source of emotional support and friendship to us [37,96,97]. As descendants of solitary hunters [96], such sociocognitive skills might be less evolved in cats compared with group-living animals such as humans and dogs, for whom social skills are necessary for survival. Studies on the social skills of cats are scarce (partly due to the methodological difficulties of testing cats in unfamiliar environments). Cats do seem to recognize emotions such as anger and happiness in people [98,99] and understand human intentions—as they follow pointing gestures [82,100] and can reproduce simple human movements [101]. However, cats are not able to interpret third party interactions [102], an ability present in social species. 

### 4.7. The Danger of Anthropomorphizing the Human–Cat Relationship 

Describing the relationship with one’s cat in terms resembling an ‘intimate and social human relationship’ (such as best friend or child) might be associated with a high tendency to anthropomorphize the cat’s behavior. Perceiving the cat as a small human can result in a miss-match between the needs of the owners and the needs of the cat. For example, humans display their affection by hugging but being held tight is threatening to a cat. Moreover, although cats enjoy petting [103], both the cat’s preferred duration and places on the body that are touched are often not compatible with the preference of the owner [104]. Additionally, owners who perceive their cat as a small human might misinterpret their cat’s (subtle) behavior or exaggerate their social abilities. Previous research showed that people’s interpretations of animal behavior are often biologically invalid [105] and that correct interpretation of facial expressions [106,107] and vocalizations of cats is difficult [108,109]. Although some degree of anthropomorphism might be necessary to experience a connection with an animal [110], owners need to keep in mind that cats are not small humans. Cats are animals, with their own abilities and needs that are different from that of humans. Our results suggest that especially owners who see their cats as best friends or children might benefit from realistic information about cats’ abilities, their natural behavior, and welfare needs. 

### 4.8. Limitations 

Several limitations need to be considered. Although we had some information about the social environment (living alone or not, perception of social support provided by the cat, and loyalty of cat versus people), we did not assess participants’ social environment in detail. For example, we had no information about whether the respondents had children due to a technical error. These questions were unfortunately not displayed when a participant indicated not to live alone. Second, our sample is not entirely representative for the Dutch cat-owner population regarding household composition, educational level, gender, number of cats in the household, and cat breed. While most cats in the Netherlands are owned by families with children [81], only one third of our respondents indicated to share their home with another person. With regard to educational level, in 2016, 31% of inhabitants of The Netherlands had a low educational level, 38% an intermediate level, and 29% were highly educated [81]. In our sample, people with a low educational level were under-represented (11%) and people with a high educational level were over-represented (54%). As is common in animal-related research, women were over-represented in our sample, despite actively encouraging men to participate. This over-representation of women in our study, combined with the following gender differences, show that our results might be less applicable to male cat owners: women have a more positive attitude towards animals [111], seem to attach more to animals [6,63,112,113], have more empathy for living beings [114], and more often attribute traits such as ‘communicative’ and ‘empathetic’ to cats [82] (this study, results not shown). Third, although participants lived all over the Netherlands, people from the northern part of the country were slightly over-represented. This is relevant, as data from 2016 [81] showed that inhabitants of the three northern provinces more often had multiple cats compared with other Dutch provinces (35% versus 27%). Fourth, respondents could not choose a relationship category related to the function of the cat as a pest controller (‘mouser’). This was intentional, as provision of food and shelter in exchange of keeping the owner’s living environment free of rats and mice can be seen as an agreement, but in our opinion, not as a relationship. These owners most likely have a dominionistic view of their cats (as suggested for dogs by Blouin [115]) where they value their cats primarily for their utility. In another study we did include the ‘mouser’ option and only 4% (*n* = 328, unpublished data) of a comparable sample of cat owners chose this option. However, including aspects of the utility role of cats in future studies may result in interesting insights on (absence of) anthropomorphic attitudes towards cats. Lastly, as is common in studies about human–animal relationships, this research unintentionally attracted highly engaged cat owners, which may have biased our results.

## 5. Conclusions

We showed in a large sample that cat owners differ in their perception of the relationship with their cat, that both owner and cat-related factors influence relationship perception, and that the living environment of the cat differs according to relationship perception. Although at this stage we have not yet formulated a complete and overarching theory to integrate all the factors that were assessed, most of our hypotheses concerning the perception of the cat and owner behaviors towards the cat were supported by the findings in our sample (for an overview see Table S1). 

The perception that the owner has of the cat’s support and dependency plays an important role in the way owners perceive the relationship with their cat. Cat owners who perceive their cats as children display similar behaviors towards their cats as human parents show towards their children, such as (over) protection and high quality (exclusive) care. Our findings suggest that owners who describe the relationship with their cats in an intimate social human role (such as best friend or child) anthropomorphize their cats to a higher extent than owners who describe the relationship in a less intimate social role (such as family member or pet). Although some degree of anthropomorphism is necessary to experience a connection with an animal, owners need to be aware that cats experience the world differently than humans and have different needs. To what extent, and in which circumstances, anthropomorphic perceptions are beneficial or detrimental to cats deserves more study. We hope that our results make owners more conscious about their own social needs and how this influences the living environment and wellbeing of their cats. 

## Figures and Tables

**Table 1:** Characteristics of owners and cats.

Owner Characteristics	*N*	%
Female	1626	91%
Educational level		
High	931	53%
Medium	613	35%
Low	199	11%
Cat professional	181	10%
Age		
<35 yrs.	555	31%
35–55 yrs.	909	50%
>55 yrs.	339	19%
Social living situation		
Alone	395	22%
With 1 other person	845	47%
With >1 other person	563	31%
Cat characteristics		
Pedigree	574	32%
Female	834	46%
Neutered	1646	91%
Cat as kitten (<3 months)	754	42%
First cat	380	21%

**Table 2:** Likelihood ratio tests.

Factor	Chi-Square	df	*p*-Value	
Owner characteristics				
Gender	3.70	3	0.296	
**Age**	**40.72**	**6**	**<0.001**	
Educational level	5.07	6	0.535	
Cat professional (yes)	3.63	3	0.304	
Owner’s social living situation				
**Presence other humans** (**yes**)	**29.57**	**3**	**<0.001**	
Presence other cats (yes)	8.31	3	0.040	
Relationship indicators				
Company	19.16	12	0.085	
**Dependency**	**38.82**	**12**	**<0.001**	
**Equality**	**76.74**	**12**	**<0.001**	
Empathy	11.96	12	0.448	
Loyalty	22.61	12	0.031	
Purpose	16.11	12	0.186	
**Support**	**39.34**	**12**	**<0.001**	
Cat characteristics				
Cat as kitten (yes)	2.20	3	0.532	
**Pedigree cat** (**yes**)	**16.44**	**3**	**0.001**	
First cat (yes)	5.24	3	0.155	
Gender cat (female)	7.96	3	0.047	
Cat’s social behavior				
Allows petting (yes)	12.21	6	0.058	
Allows lifting (yes)	5.64	9	0.775	
Jumps on lap (yes)	3.81	3	0.450	

**Bold**: Significant factors associated with relationship perception category.

## Data Availability

The data presented in this study are openly available in DataverseNL at https://doi.org/10.34894/OXZRWO.

## Supplementary Materials

The following supporting information can be downloaded at: https://www.mdpi.com/article/10.3390/ijerph19010193/s1, Figure S1. Distribution of respondents over the Netherlands, Table S1a. Hypotheses regarding owner and cat characteristics, Table S1b. Hypotheses regarding relationship aspects, Table S1c. Hypotheses regarding the cat’s living environment, Table S2. Descriptives by group.
