# Supplementary material for: Family Member, Best Friend, Child or ‘Just’ a Pet, Owners’ Relationship Perceptions and Consequences for Their Cats
Source: Int J Environ Res Public Health. 2021 Dec 24;19(1):193. doi: 10.3390/ijerph19010193 (PMC8750854; doi:10.3390/ijerph19010193)
Supplement: Supplementary file 1 [file ijerph-19-00193-s001.zip › ijerph-1448728-supplementary.pdf]

Supplementary figures and tables

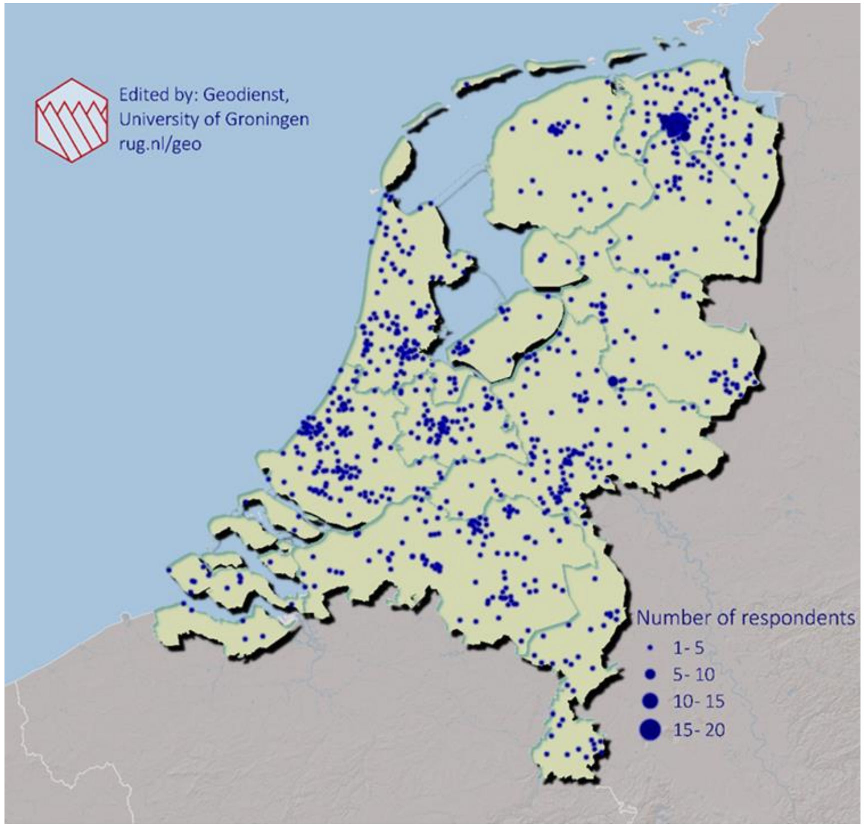

Figure S1. Distribution of respondents over the Netherlands

Table S1a. Hypotheses regarding owner and cat characteristics

	Gender	Age group*	Owners social living situation*	Pedigree*
Family member			family, couple	
Child	women		**single**	**likely**
Best friend	men		**single**	
Pet	men	**older**		**no**

Table S1b. Hypotheses regarding relationship aspects

	Equal to family*	Empathetic	Loyal	Dependent on owner for love and care*	Important source of support and companionship*
Family member	yes			**to some extent**	no
Child	no, authoritarian	yes		**yes**	**yes**
Best friend	**yes**	yes	yes	to some extent	**yes**
Pet	**no**	no	no	**hardly**	**no**

Table S1c. Hypotheses regarding the cat's living environment

	Bedroom access**	Sleep on bed with owner	Care in owner's absence**	Outdoor access**
Family member	yes			**yes**
Child	**yes**		boarding/prof cat sitter	**no or restricted**
Best friend	**yes**	yes		yes
Pet	**no**	no	neighbors/friends	**yes**

Notes: * Significant determinants for relationship perception, ** Significantly different between relationship categories, **Bold**: hypothesis confirmed by results.

Table S2. Descriptives by group

		Family		Child		Best friend		Pet		Total sample		Group difference
**Owner characteristics**		**n**	**%**	**n**	**%**	**n**	**%**	**n**	**%**	**n**	**%**	**X2 (df), p-value**
Gender	Female	857	91	468	95	96	88	205	81	1626	91	X2 (1797,3) = 42.41, <0.001
Age	< 35	254	27	218	44	33	30	50	19	555	31	X2 (1803,6) = 66.36, <0.001
	35-55	505	54	206	42	54	50	144	56	909	50	
	> 55	184	20	69	14	22	20	64	25	339	19	
Education	low	96	11	63	13	22	21	18	7	199	11	X2 (1747,6) = 55.09, <0.001
	middle	306	33	201	43	43	41	63	25	613	35	
	high	515	56	206	44	40	38	170	68	931	53	
Cat professional	yes	97	10	53	11	15	14	16	6	181	10	X2 (1788,3) = 6.34, 0.10
**Owners' social environment**		**n**	**%**	**n**	**%**	**n**	**%**	**n**	**%**	**n**	**%**	**X2/F (df), p-value**
Living alone	yes	156	17	146	39	46	42	47	19	395	22	X2 (1799,3) = 30.99, <0.001
Presence of cats	one	342	36	140	28	48	44	123	48	653	36	X2 (1803,3) = 61.27, <0.001
	two	340	36	206	42	31	28	93	36	670	37	
	three	125	13	77	16	11	10	29	11	242	14	
	> three	133	14	70	14	19	17	12	5	234	13	
Average # cats (mean, sd)		2.3	1.8	2.4	1.7	2.3	1.9	1.9	1.7	2.3	1.8	F (1798,3) = 4.46, 0.004
**Cat characteristics**		**n**	**%**	**n**	**%**	**n**	**%**	**n**	**%**	**n**	**%**	**X2 (df), p-value**
Pedigree	yes	293	31	203	41	35	32	43	17	574	32	X2 (1803,3) = 47.44, <0.001
	British Shorthair	74	25	51	25	10	29	11	26	146	25	
	Ragdoll	67	23	49	24	8	23	5	12	129	23	
	Maine Coon	34	12	23	11	4	11	7	16	68	12	
	Siamese	21	7	16	8	4	11	4	9	45	8	
	Norw. Forest Cat	13	4	6	3	2	6	1	2	22	4	
	Persian	10	3	5	3	2	6	1	2	18	3	
	Other breed	72	25	53	26	5	14	14	33	144	25	
Age	< 5 yrs	388	41	267	54	43	40	102	40	800	44	X2 (1802) = 29.23, <0,001
	5-10 yrs	297	32	127	26	35	32	78	30	537	40	
	10-15 yrs	189	20	73	15	23	21	53	21	338	19	
	> 15 yrs	69	7	26	5	7	7	25	10	127	7	

As kitten	yes	409	43	203	41	37	34	105	41	754	42	X2 (1803,3) = 3.93, 0.269
First cat	yes	173	18	129	26	20	18	58	23	380	21	X2 (1803,3) = 12.70, 0.005
Sex cat	female	430	46	237	48	37	34	130	50	834	46	X2 (1803,3) = 9.24, 0.026
Neutered	yes	851	90	452	92	100	92	243	94	1646	91	X2 (1803,3) = 4.14, 0.246
**Cat's social behaviour**		**n**	**%**	**n**	**%**	**n**	**%**	**n**	**%**	**n**	**%**	**X2 (df), p-value**
Allows stroking	often/always	913	97	465	94	105	96	238	92	1721	96	X2 (1797,3) = 18.37, <0.001
Allows lifting	often/always	733	89	399	84	88	60	185	72	1409	78	X2 (1803,3) = 11.81, 0.008
Voluntary on lap	yes	741	79	387	79	78	72	197	76	1403	78	X2 (1803,3) = 3.24, 0.356
**Cats' environment**		**n**	**%**	**n**	**%**	**n**	**%**	**n**	**%**	**n**	**%**	**X2 (df), p-value**
Outdoor access	yes	643	68	258	52	65	60	204	79	1217	66	X2 (3,1803) = 62.71, <0.001
How	Catflap always open	194	30	57	22	22	34	83	41	369	30	X2 (6,1170) = 24,87, <0.001
	Catflap time restricted	70	11	34	13	8	12	9	4	125	10	
	Let in/out by person	379	59	167	65	35	54	112	55	723	60	
Where	Everywhere	409	66	115	46	29	47	159	83	741	64	X2 (9,1120) = 79,00, <0.001
	Balcony	33	5	29	12	4	6	9	5	82	7	
	Fenced yard	148	24	84	34	22	36	22	11	282	24	
	Catio	28	5	20	8	7	11	2	1	57	5	
Access to bedroom	yes	490	52	355	72	78	72	94	36	1017	57	
Sleeps on bed	yes	423	87	319	90	70	89	75	80	887	88	
Care when away	Paid sitter	46	5	23	5	5	5	9	4	87	5	X2 (23,1753) = 159,23, <0.001
	Boarding	37	4	17	4	4	4	9	4	68	4	
	Friends/neighbours	663	71	326	67	66	61	199	78	1292	71	
	Combination of options	85	9	36	7	5	5	15	6	148	8	
	Never absent	98	11	82	17	27	25	21	8	233	13	
**Relationship indicators**		**mean**	**sd**	**mean**	**sd**	**mean**	**sd**	**mean**	**sd**	**mean**	**sd**	**F (df), p-value**
Company		4.5	0.6	4.7	0.5	4.5	0.7	4.2	0.8	4.5	0.6	F (3,1788) = 32.06, < 0.001
Dependency		3.0	1.1	3.8	1.1	3.5	1.2	2.3	1.0	3.1	1.2	F (3,1767) = 101.89, < 0.001
Empathy		3.5	1.0	4.0	1.0	4.0	1.0	2.9	1.1	3.6	1.1	F (3,1717) = 68.22, < 0.001
Equality		2.7	1.1	3.5	1.2	3.4	1.2	1.9	0.9	2.9	1.3	F (3,1744) = 120.42, < 0.001

Loyalty	3.3	1.1	4.1	1.0	4.1	0.9	2.6	1.0	3.5	1.1	F (3,1752) = 138.18, < 0.001
Purpose	3.1	1.1	3.8	1.1	3.8	1.0	2.4	1.0	3.3	1.2	F (3,1796) = 111.15, < 0.001
Support	4.0	0.8	4.5	0.7	4.5	0.6	3.3	1.1	4.1	0.9	F (3,1773) = 155.32, < 0.001
